# Application of Thin-Layer Chromatography-Flame Ionization Detection (TLC-FID) to Total Lipid Quantitation in Mycolic-Acid Synthesizing *Rhodococcus* and *Williamsia* Species

**DOI:** 10.3390/ijms21051670

**Published:** 2020-02-29

**Authors:** Akhikun Nahar, Anthony L. Baker, David S. Nichols, John P. Bowman, Margaret L. Britz

**Affiliations:** 1 Tasmanian Institute of Agriculture, University of Tasmania, Hobart, TAS 7005, Australia; akhikun.nahar@utas.edu.au (A.N.); a.l.baker@utas.edu.au (A.L.B.); john.bowman@utas.edu.au (J.P.B.); 2 Central Science Laboratory, Division of Research, University of Tasmania, Hobart, TAS 7005, Australia; d.nichols@utas.edu.au

**Keywords:** TLC-FID, Iatroscan rods, NMR, lipid extraction, mycolic acids, *Williamsia*, *Rhodococcus*

## Abstract

In addition to cell membrane phospholipids, *Actinobacteria* in the order *Corynebacteriales* possess a waxy cell envelope containing mycolic acids (MA). In optimized culture condition, some species can also accumulate high concentrations of intracellular triacylglycerols (TAG), which are a potential source of biodiesel. Bacterial lipid classes and composition alter in response to environmental stresses, including nutrient availability, thus understanding carbon flow into different lipid classes is important when optimizing TAG synthesis. Quantitative and qualitative analysis of lipid classes normally requires combinations of different extraction, derivatization, chromatographic and detection methods. In this study, a single-step thin-layer chromatography-flame ionization detection (TLC-FID) technique was applied to quantify lipid classes in six sub-Antarctic *Corynebacteriales* strains identified as *Rhodococcus* and *Williamsia* species. A hexane:diethyl-ether:acetic acid solvent system separated the total cellular lipids extracted from cells lysed by bead beating, which released more bound and unbound MA than sonication. Typical profiles included a major broad non-polar lipid peak, TAG and phospholipids, although trehalose dimycolates, when present, co-eluted with phospholipids. Ultra-performance liquid chromatography-tandem mass-spectrometry and nuclear magnetic resonance spectroscopy detected MA signatures in the non-polar lipid peak and indicated that these lipids were likely bound, at least in part, to sugars from cell wall arabinogalactan. Waxy esters were not detected. The single-solvent TLC-FID procedure provides a useful platform for the quantitation and preliminary screening of cellular lipid classes when testing the impacts of growth conditions on TAG synthesis.

## 1. Introduction

Lipids are important cellular components of biological systems and are involved in membrane formation of cells and cellular structures, signaling and energy storage [1]. Membrane lipids are mainly phospholipids (PL) in the form of two fatty acid (FA) chains linked to glycerol–phosphate derivatives, where the structures vary amongst bacterial species. Bacterial membrane lipid composition can change in response to altered environmental conditions, aiding survival [2] and maintaining appropriate membrane viscosity which influences other membrane-related functions, including transport of hydrophobic molecules and solutes, and protein–protein interactions [3]. In addition to PL, members of the order *Corynebacteriales* synthesize very long-chain lipids in their cell wall, the α-alkyl β-hydroxy fatty acids termed mycolic acids (MA) [4,5]. Along with cell wall MA, some species of this group can accumulate triacylglycerols (TAG) as storage lipids, which are considered as potential substrates for biofuel development [6]. The most studied lipid-accumulating genus is *Rhodococcus*, which contains many species considered to have industrial potential for TAG synthesis [7,8,9,10,11]. An important parameter in optimizing bacterial TAG production is the efficient flow of carbon sources provided in growth media into the target product, as well as cell biomass. However, quantitative monitoring of the distribution of carbon into the suite of storage and structural lipids (PL, MA and other surface lipid structures) is hampered by the lack of a single method to quantify all of the lipids.

Bacterial lipid composition has been used for taxonomy since 1963 [12,13] and lipid profile analysis, termed “lipidomics”, along with proteomics and genomics, is important for understanding metabolic processes [1,14]. Advances in mass spectrometry (MS), coupled with different chromatographic techniques, have facilitated highly sensitive and accurate analysis of different lipid classes from various biological systems [15]. However, not all the lipid classes are amenable to direct detection by ion fragmentation techniques of MS. The most common technique used for measuring membrane and storage lipids is gas chromatography-mass spectrometry (GC-MS), which usually analyzes fatty acid methyl esters (FAME) produced by saponification of complex lipids to liberate FA from PL, TAG and other lipid classes, followed by esterification. Analysis of FAME is useful for taxonomic purposes, but it does not provide information on the lipid classes present or whether they are storage or structural lipids [16]. Ionization techniques have developed further to enable direct analysis of lipid classes, including electrospray ionization (ESI), matrix-assisted laser desorption ionization (MALDI) and fast atom bombardment ionization for non-volatile lipids [17,18,19]. However, accurate quantification of lipid classes is not feasible due to the requirement of ^13^C-labeled standards for differing lipid classes [20].

MAs are one of the major lipid classes in *Corynebacteriales*, constituting up to 60% of the cell walls of mycobacterial species [21] and can occur as solvent extractable free mycolic acids (‘unbound’) or covalently bound to cell wall structures, notably trehalose, glycerol or arabinogalactan [22,23,24]^.^ Free MA and their derivatives, including methyl esters (MAME) or trimethylsilyl (TMS) ether derivatives of the methyl esters, have been used to identify MA structures and distinguish different *Mycobacterium* species as well as other MA-synthesizing genera using different chromatographic-MS techniques [25,26,27,28]. However, it is often difficult to analyze the esters of MA by GC-MS, particularly high molecular weight species, as this requires very high temperatures to elute MA molecules [29]. Quantification is more complicated due to the lack of availability of commercial standards for the diverse chemical structures that make up MA. Over 500 related chemical structures have been identified from *M. tuberculosis* alone [30].

The composition of *Corynebacteriales* cell envelopes varies with genus and, sometimes, between species within a genus. The most complex cell envelopes are found in the *Mycobacterium* species, typified by *M. tuberculosis* and related pathogenic and environmental strains, with long-chain MAs of up to 90 carbons in length. In contrast, *Corynebacterium* species contain the smallest chain lengths of C_22_–C_38_ and *Gordonia*, *Rhodococcus* and *Williamsia* species contain medium chain length MAs of around C_30_–C_60_ [22]. *Corynebacteriales* cell walls contain various lipid species and possess an outer membrane which acts as a permeability barrier. Qualitative analysis of outer membrane lipids is challenging, as conventional mechanical and/or chemical lipid extraction processes may result in a mixture of cytoplasmic membrane lipids (inner membrane) and possibly storage lipids released by lysis [23,25,31,32]. Despite a long history of research characterizing the makeup of the cell envelopes of the *Corynebacteriales* members, both the models of the structures and methods to characterize the components are still evolving [5,33,34]. Bansal-Mutalik and Nikaido [35,36] developed a method to extract separately the outer and inner membrane lipids from *C. glutamicum* and *M. smegmatis* cells using reverse surfactant micelles and found that the outer membrane is mostly composed of covalently bound MA. The inner membrane contains a small amount of MA in the form of trehalose mono-mycolate (TMM) plus PL typically found in the cell membrane. However, together with MA, *C. glutamicum* produces a large amount of FA (C_12_–C_20_) in the form of peptidoglycan-embedded cardiolipin in the outer membrane, while the *M. smegmatis* outer membrane contained TAG, DAG and glycopeptidolipids, plus yet-uncharacterized non-polar lipids. Consequently, measuring storage lipids by quantifying FAME may include FA originating from intracellular storage vesicles (TAG), membrane PL and outer membrane lipids (TAG, PL and free FA). Reverse surfactant micelle methods have not yet been applied to *Rhodococcus* or *Williamsia* species so the make-up of the outer membrane in these genera is not well documented, although the MA content in rhodococci has been extensively characterized [28,37].

As part of a broader project evaluating environmental *Corynebacteriales* strains for potential TAG synthesis, we were interested in developing rapid screening methods to quantify and differentiate the major lipid classes synthesized under different culture conditions. Thin-layer chromatography-flame ionization detection (TLC-FID) had been used locally for quantifying lipids in marine environmental and food samples [38,39], and well-established procedures, while less sensitive than GC-MS, can reliably quantify and differentiate between TAG, PL and potential breakdown products of TAG when operated under optimized conditions [40,41,42,43,44]. The aim of the current study was to determine whether this simple, comprehensive and rapid method of screening neutral and polar lipids could be applied to MA-producing bacteria, as a preliminary approach to screening and quantifying lipid classes prior to quantifying total FA by GC-MS. A single method that can detect, separate and quantify MA (free and bound), TAG, PL and FA in a mixture has not been described previously.

## 2. Results

### 2.1. Separation of Lipid Standards and Bacterial Extracts Using Plate TLC

**Figure 1:**
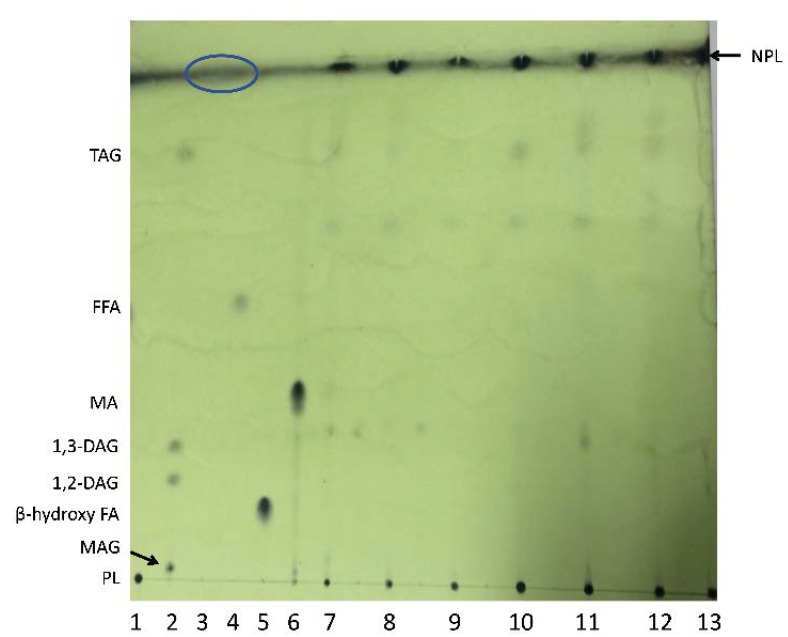
Thin-layer chromatography (TLC) plate separation of bacterial lipids extracted from whole cells using chloroform:methanol (unbound MA extract), developed with the H:D:F 70:30:0.2 solvent system. Lanes correspond to: 1. PL; 2. MAG, DAG, TAG (Supelco lipid standard 1787-1AMP); 3. HC (blue circle at the top of the plate shows the diffuse area where this standard typically ran); 4. FFA; 5. β-hydroxy FA; 6. MA (mycobacterial free MA standard); 7. *Williamsia* sp. 1135; 8. *Williamsia* sp. 1138; 9. *Rhodococcus qingshengii* 1139; 10. *Rhodococcus erythropolis* 1159; 11. *Rhodococcus* sp. 1163; 12. *Rhodococcus* sp. 1168; 13. *C. glutamicum*. NPL = Non-polar lipid spots in bacterial extracts running with the solvent front at the top of the plate.

To confirm the presence of MA, TAG and FFA in bacterial extracts, lipid extracts of six sub-Antarctic strains and the *Corynebacterium glutamicum* control were prepared by bead beating freeze-dried cells in chloroform:methanol (2:1, *v/v*), then the extracted lipids and commercial standards were separated on silica-TLC plates. The solvent system selected for this initial screening was hexane:diethyl-ether:formic acid (H:D:F) (70:30:0.2, *v/v/v*) which was reported by Striby et al. [41] to resolve TAG, FA and diacylglycerol isomers by TLC-FID. This solvent resolved TAG, 1,2- and 1,3-diacylglycerols (DAG), monoacylglycerols (MAG), β-hydroxy-fatty acid and the free MA (mycobacterial) standards on TLC plates (Figure 1). However, the PL standard remained at the origin and the hydrocarbons (HC) standard typically ran with the solvent front and spread over a large area. The presence of TAG was observed in bacterial extracts, except for the *C. glutamicum* control, as expected. The bacterial extracts all showed distinct major lipid spots at the solvent front which were well separated from the free MA standard, plus minor lipid spots that did not correspond to the migration distance of any standard. We had anticipated that any unbound MA extracted from the test strains would migrate with *R_f_* values similar to the free MA standard, but this was not observed. Consequently, the nature of these lipids, particularly the major lipid detected at the solvent front, was further evaluated by TLC-FID and other analyses, as described below.

### 2.2. Separation of Standards and Lipids in Bacterial Extracts by TLC-FID

**Figure 2:**
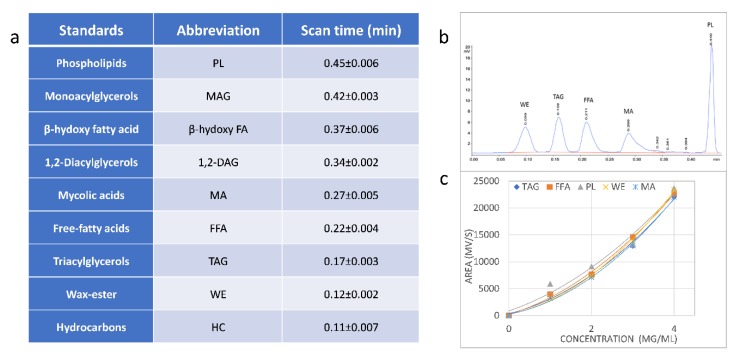
Separation of commercial standards on Iatroscan rods developed with the H:D:A (80:20:0.1, *v/v/v*) solvent and detection by FID scan. Panel (**a**) shows the average scan times (min) and the standard deviations for 3–5 repeated applications of each compound and the abbreviations used in panels (**b**) and (**c**); (**b**) is an example of the separation of selected standards loaded onto rods at 1 mg/mL each, chromatogram plotting response (mV) against scan time (min); (**c**) an example calibration curve for selected compounds for loadings between 1 to 4 mg/mL each. PL, phospholipid, and trehalose dimycolate, TDM, ran with similar scan times. The mycobacterial MA standard showed a broad, tailing peak in panel (**b**), indicating heterogeneity in this preparation.

Lipid analysis by TLC-FID typically involves using multiple solvents to develop Iatroscan rods, so that non-polar lipids are separated, scanned by FID, then the rods are re-developed in one or more polar solvents to differentiate the remaining lipids and pigments [40,41,42,43,44]. As a starting point for TLC-FID using silica-coated quartz Iatroscan rods, we trialed a less polar solvent than used in plate TLC, hexane:diethyl-ether:acetic acid (H:D:A) (70:10:0.1, *v/v/v*), to resolve lipids in the region of migration of TAG and the major non-polar lipids seen on plates. Although optimal hydrogen and air flow conditions for the Iatroscan MK-6 instrumentation were used in conjunction with rapid scan times (0.5 min/rod) [44], MAG, 1,2-DAG and β-hydroxypalmitic acid were not resolved. Another H:D:A system (80:20:0.1, *v/v/v*) (based on [43]) was then used in a single development and this showed a clear separation of MAG, 1,2-DAG, TAG, FFA, β-hydroxy palmitic acid, wax-ester (WE), HC and PL in the standards (Figure 2a,b). The commercially supplied free mycolic acid standard formed a broad peak with scan times between 0.25 and 0.33 min, indicating heterogeneity in the preparation, as expected from the size distribution of 60 to 90 carbons in mycobacterial MA (from the Sigma-Aldrich technical data sheets). Trehalose dimycolate (TDM) and PL had similar TLC-FID scan times at 0.45 ± 0.006 min. Given the observed clear separation of non-polar lipids (WE, HC), TAG, FFA and 1,2-DAG, further solvent optimization was not pursued. The scan times were reproducible between runs on single batches of rods, as indicated from the standard deviations shown in Figure 2a, and calibration curves were constructed using up to 10 mg/mL of individual standards. While showing linear peak area responses at higher concentrations, broad peaks were observed, and baseline resolution was lost if a mixture of standards was applied to rods at a total lipid concentration >25 mg/mL. A mixture of five lipid standards (representing the major lipids classes later found in cell extracts) was routinely used to produce calibration curves for standards in the range of 1–4 mg/mL (Figure 2c shows a typical set of curves for these selected standards which follow the power law y = ax^b^, as reported previously [41]). Similar loadings of standards have been used previously [41,42,43,44] and with the same TLC-FID instrumentation [44], with reported limits of sensitivity of 0.2 mg/mL depending on the FID response for each standard [43].

**Figure 3:**
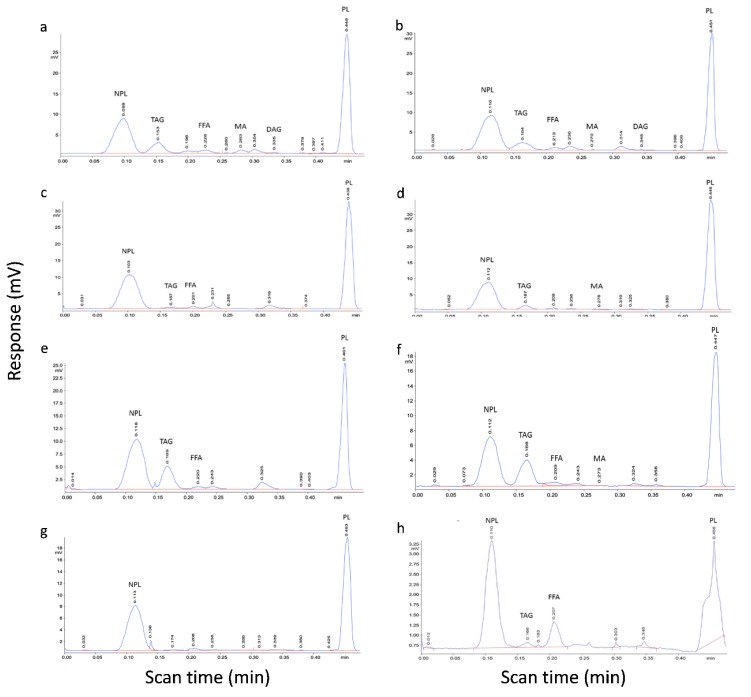
Typical TLC-FID chromatograms obtained using the H:D:A (80:20:0.1, *v/v/v*) solvent development system for unbound lipid extracts of sub-Antarctic strains and controls. (**a**) *Williamsia* sp. 1135; (**b**) *Williamsia* sp. 1138; (**c**) *Rhodococcus qingshengii* 1139; (**d**) *Rhodococcus erythropolis* 1159; (**e**) *Rhodococcus* sp. 1163; (**f**) *Rhodococcus* sp. 1168; the control strains, (**g**) *C. glutamicum* and (**h**) *M. phlei*. The large non-polar lipid (NPL) peak was observed in the chromatogram of all the bacterial strains. Chromatograms are marked with the scan times corresponding to the lipid standards when equivalent peaks were detected in the bacterial extracts in a final volume of 500 µL (see Figure 2 for abbreviations and Section 4.2 for methods).

All six bacterial extracts and the control strains, *M. phlei* and *C. glutamicum*, showed major peaks with scan times corresponding to PL/TDM and a non-polar lipid (NPL) which had a scan time similar to the WE and HC standards (Figure 3). *M. phlei*, like other mycobacterial species, synthesizes TDM as a major component of its lipid layers, so if significant amounts of TDM were present in extracts this would be seen as a split or broader peak in the PL region. Other detected lipids corresponded to TAG, FFA and 1,2-DAG: strains 1135, 1138 (*Williamsia* spp.) and 1163, 1168 (*Rhodococcus* spp.) showed proportionately greater TAG synthesis under the growth conditions used. There was a small quantifiable peak corresponding to the scan time of the free MA standard at 0.27 min in extracts of *Williamsia* sp. 1135. For the other strains, very minor peaks were observed at this scan time and the area was <1% of the total area measured by FID. Several of the bacterial extracts also had a minor peak in the region of 0.300–0.325 min which was distinct from the scan time seen for the 1,2-DAG standard and less than the scan time for the free MA standard from bovine *M. tuberculosis*. These observations were generally similar to results seen by plate TLC (Figure 1). The minor peaks could correspond to 1,3-DAG, given that the scan time relative to the other standards used for TLC-FID eluted in the same order as seen for this compound in the commercial standard lipid mixture for TLC (Figure 1, lane 2). However, a suitable standard for pure 1,3-DAG was not available for TLC-FID analysis and standards were made up from individual compounds. Evidence from saponified extracts for bound lipids (see Section 2.4) may also suggest that this minor peak corresponds to free MA. Bacterial lipid classes were confirmed by mixing individual standards with bacterial extracts and observing co-elution (Supplementary Figure S1).

### 2.3. Identification of Lipids in the Non-Polar Lipid Peak

As the scan time for the non-polar lipid peak coincided with HC and WE standards, GC-MS analysis was performed to determine whether WE was present in the bacterial extracts by analysis for non-saponifiable (HC) or saponifiable (alcohol) neutral lipids that are signatures of WE complex lipids, as described in Materials and Methods Section 4.5. The full-scan total ion chromatograms of the non-saponifiable lipid bacterial extracts (Supplementary Figure S2) exhibited peaks, but these were determined to be aldehyde compounds (octadecanal, docosanal, tetracosanal and hexacosanal) in *R. erythropolis* strain 1159 and not HC or TMS-alcohols, which would have been released from the saponification of WE if present in the extracts. These data indicated that WE were not present in the bacterial lipid extracts.

**Figure 4:**
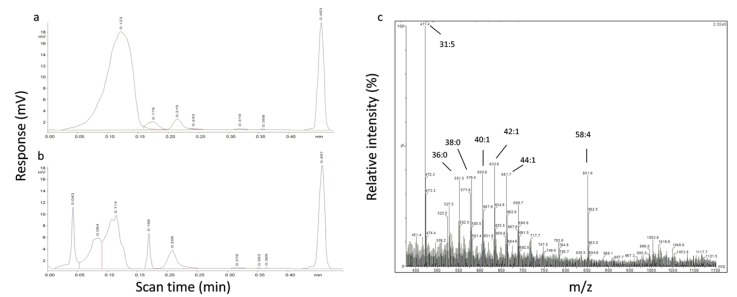
TLC-flame ionization detection (FID) chromatogram of bacterial total lipid extract from *Williamsia* sp. 1138 (**a**) before methanol wash of the Iatroscan rod; (**b**) after eluting the non-polar peak region with methanol from the Iatroscan rod. Panel (**c**) shows the electrospray ionization (ESI)-MS full-scan mass spectrum of the methanol-eluted, non-polar lipid material with [M-H]^−^ ions (m/z) corresponding to MA and the carbon chain lengths of some major MA marked.

To further explore the chemical nature of the lipids in the non-polar lipid peak detected in TLC-FID, a total lipid extract from *Williamsia* sp. 1138 was applied to six Iatroscan rods and the NPL peaks collected by washing with methanol following separation using the H:D:A solvent (see Section 4.6). The washed rods were dried and rescanned to check that the washing procedure was efficient. A loss of peak area of around 50% for the non-polar lipid area was observed after rescanning the washed rods (Figure 4a,b). Similar results were obtained in repeated experiments for strain 1138 and with strain *R. qingshengii* 1139. The pooled and concentrated methanol washes were analyzed by UPLC-MS/MS in negative electrospray ionization (ESI) mode. The carbon chain lengths for ions corresponding to MA were C_31_ to C_58_ as determined from ESI-MS full scan mass spectra (Figure 4c), confirming the presence of MA in the eluted material.

**Figure 5:**
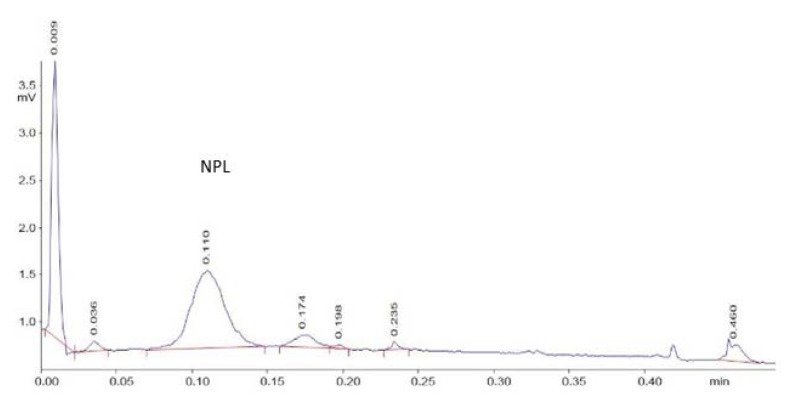
TLC-FID chromatogram of the scraped TLC plate spot extract of *R. qingshengii* 1139, showing the NPL as the major peak at the expected scan time 0.11 min.

The concentration of lipids in the methanol extracts from the Iatroscan rods was not, however, high enough for NMR analysis. Consequently, lipid extracts of *R. qingshengii* 1139 were loaded onto multiple lanes of TLC plates, using the methanol extract from strain 1138 as the control to determine the migration distance of the non-polar lipid. Strain 1139 was selected in these experiments, as TAG production was relatively less than the other strains (Figure 3) so possible contamination with TAG was minimized. After developing the lane with the rod-washed lipid, silica gel was scraped from the equivalent region of the plate for the bacterial extracts. The concentrated, eluted lipid extract from the TLC plates was run on Iatroscan rods to confirm that this contained non-polar lipid, which was detected as the major peak present at the expected scan time (Figure 5). This ‘plate wash’ extract was then subjected to NMR analysis.

**Figure 6:**
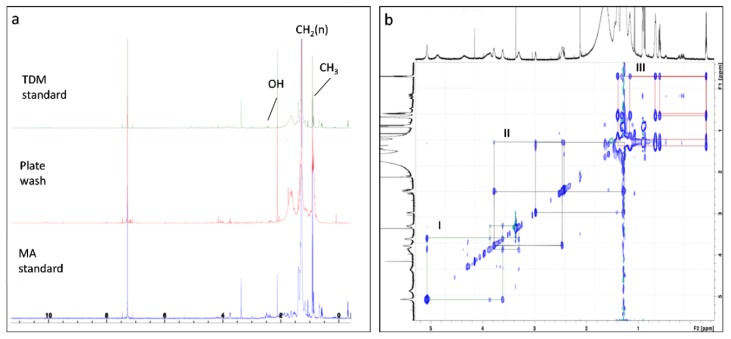
NMR spectra of trehalose dimycolate (TDM) standard, unknown lipid extracted from Iatroscan rods and free mycobacterial MA standard, showing the characteristics resonances of MA including long aliphatic chain (CH_2_[n]) at δ 1.0 ± 1.5 ppm (peak at 1.3), terminal methyl group at δ 0.86 ppm and hydroxyl group at ca. δ2.5 ppm (**a**) and combined image from two plots of the TDM standard by TOCSY analysis, showing both high and low abundance components in the one image (**b**). Each color in (b) represents a separate molecular system. The green system (I) shows the characteristic resonance of sugar molecules, the black system (II) represents an acyl chain linked to CH_2_ signals around 1.3 ppm and the red (III) system is representing a distinct system containing *cis-*cyclopropyl signatures in methylene chains.

The 600 MHz ^1^H-NMR spectra of free MA and TDM standards from *Mycobacterium*, and the non-polar lipid material, are shown in Figure 6a. A very strong peak was found for long aliphatic chains at δ 1.3 ppm and the terminal methyl group at δ 0.86 ppm, plus a minor peak at ca. δ 2.5 which is characteristic of the β-hydroxyl group in MA [45], in both standards and the unknown lipid. These resonances of the unknown lipid and its similarities with MA and TDM standards indicated that this lipid contained MA, as indicated by ESI-MS.

TOCSY analysis was performed for the TDM standard to show both high and low abundance components in the one image (Figure 6b), to verify the systems corresponding to the signatures seen in the standards and plate-washed material. There were three molecular systems (Figure 6b, colored as green (I), black (II) and red (III)) found in the TDM. The green system (I) is likely to contain sugar structures with a characteristic anomeric proton shift around 5.1 ppm, corresponding to the distinctive chemical shift seen for protons in trehalose (reference spectrum HMDB00975); other chemical shifts for protons in trehalose (3.4–3.9 ppm) were less distinct. The black system (II) corresponds to an extended acyl chain and linked back to the large signal corresponding to the bulk of the CH_2_ signals around 1.3 ppm. Conceivably, this could be mero-mycolate chains of MA, since they possess an extended α-alkyl side chain originating from FA. The red system (III) represents a highly abundant species in both the TDM and mycobacterial MA standards with some highly shielded protons in the region <0.86 ppm: this corresponds to the signals expected for *cis*-cyclopropyl structures as described in MA from *M. smegmatis* [45]. Similar signals were not observed for the plate-washed material, which is consistent with the observed absence of *cis*-cyclopropyl structures in the *Rhodococcus* and *Williamsia* MA.

**Figure 7:**
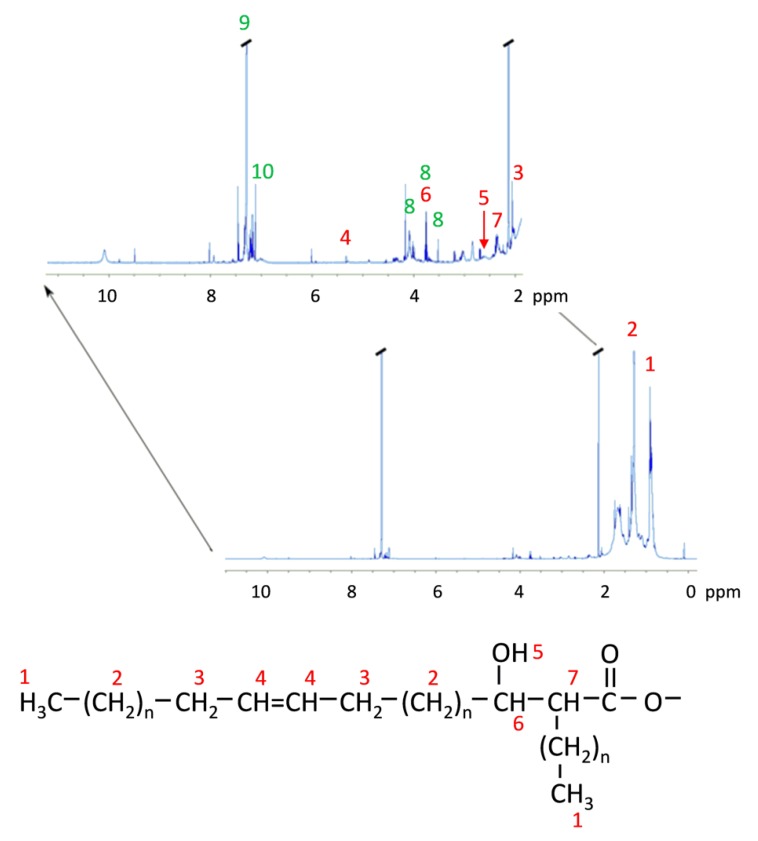
NMR spectrum for plate-washed material with an expanded scale from 2–11 ppm. Chemical shifts likely to correspond to protons in mycolic acid structures [45] are labelled in red. Peaks labelled in green are: 8, putative sugar signals; 9, CDCl_3_; 10, signals in unidentified structures. Where two labels occur, the chemical shifts for the peaks are similar for protons in sugar and mycolic acid structures.

Figure 7 shows an expansion of the scale for the NMR image in Figure 6 plate-washed material, for the region >2–11 ppm, to allow the inspection of minor peaks. Assigning potential structures associated with the chemical shifts is problematic, given that many of these overlap with resonances expected for protons in several possible structures, notably for potential sugar signals and the β-carbon proton (ca. δ 3.75 ppm), are very low signals and occur as clusters of signals. However, many of these aligned with chemical shifts reported for mycobacterial signature structures, including protons on the α-carbon and adjacent to double bonds. A cluster of multiple signals in the region 3.5–4.1 ppm corresponded to resonances expected for protons in D-galactose (reference spectrum HMDB00143), L-arabinose (HMDB00646), rhamnose (HMDB00849) and D-mannose (HMDB00169), but with undetectable signals in the region ca. 5.2 ppm found in trehalose, D-mannose and, to a lesser extent, L-arabinose. This suggests that the plate-washed material may contain sugars, particularly D-galactose and L-arabinose. Given that the lipid extracts from the bacterial strains were prepared by extensive bead beating and solvent extraction without further derivatization, we suggest that MAs remaining linked to arabinogalactan fragments, or other cell wall structures, contribute to the broad peak routinely detect by TLC-FID in all of the sub-Antarctic, and control strain, extracts. The sugar components formed a very minor part of the structures, given the low signals seen for the associated chemical shifts. A cluster of chemical shifts was also observed in the >7 ppm, around the CDCl_3_ peak; this was not further investigated, but it is noted that similar shifts occur for protons in aromatic structures.

### 2.4. Comparison of the Efficiency of Bead Beating with Sonication and Quantification of Bacterial Lipid Classes

**Figure 8:**
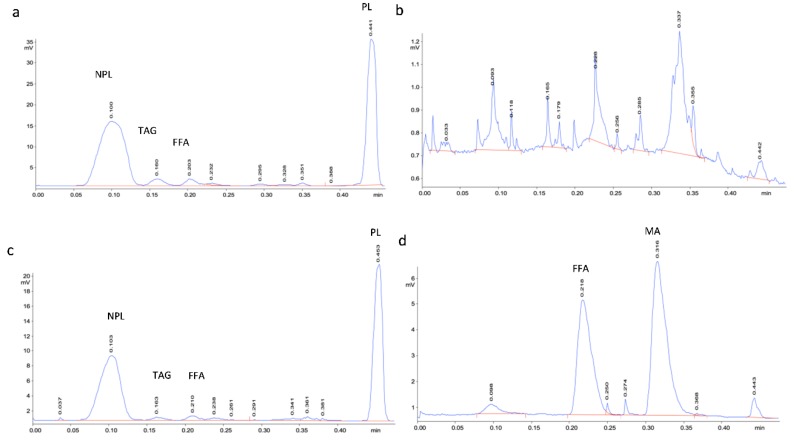
TLC-FID chromatograms of bacterial unbound and bound lipid extracted using two cell lysis methods for strain *R. qingshengii* 1139. Panels correspond to: (**a**) unbound extract after bead beating; (**b**) bound extract after bead beating; (**c**) unbound extract after sonication; (**d**) bound extract of sonication. The bound lipids (b and d) were released from cell debris by saponification with KOH following the extraction of unbound lipids (see Section 4.2). The final volume of extracts from 20 mg (dry weight) of cells was 250 µL.

Lipids were extracted from *R. qingshengii* 1139 cell by bead beating or sonication in the presence of chloroform:methanol (2:1) and analyzed by TLC-FID (Table 1, Figure 8), which would normally release the ‘unbound’ lipids. The relative proportions of the major lipid classes following both treatments were similar, with major peaks corresponding to PL/TDM and non-polar lipid plus relatively minor peaks of FFA and TAG. However, the total peak area obtained for unbound extracts following bead beating of 20 mg (dry weight) of cells was almost double that of sonication. Saponification of the cell debris pellet to release residual bound lipids following bead beating constituted 1.16% of the total peak area seen for the unbound lipid extract, indicating that little lipid was further released by saponification. In contrast, there were several lipid peaks detected following saponification after cell lysis by sonication, and these peaks represented 43% of the unbound extract based on total peak areas (Figure 8d), and around 51% based on total weight, of major lipid classes (Table 1). The scan time identified one of the major peaks as FFA (approximately 53% of total lipid by weight) and the other, very minor, peaks corresponded to scan times for the WE, MA and PL/TDM standards. The other major peak had a scan time close to the 1,2-DAG standard but was clearly distinct. This may have corresponded to 1,3-DAG potentially resulting from partial saponification of TAG, but without a standard for pure 1,3-DAG it is not possible to assign identity based on scan time. Furthermore, TAG constituted only a small proportion of the lipids detected in *R. qingshengii* 1139 cells, so it is unlikely that partial saponification of residual TAG in the cell debris after extraction of unbound lipids would yield such a proportionately large peak. As saponification with KOH would liberate FFA from acylglycerols (including PL) and would free MA from TMM, TDM and other glycolipids in the cell debris after extracting unbound lipids, we suggest that the second major peak may correspond to liberated MA. The difference in scan times between this peak and the mycobacterial MA standard may arise due to the difference in the number of carbons (ca. 60–90) and variety of functional groups in the mycobacterial standard and the bacterial extracts (31–58 carbons, Figure 4c), making the latter less hydrophobic and with a longer scan time. The MA standard typically showed a broad, tailing peak which covered the region of the peak seen at 0.300–0.325 min. This is consistent with the detection of minor peaks in the scan time region of 0.30–0.32 min in earlier TLC-FID chromatograms (Figure 3 and Supplementary Figure S1).

As bead beating released more lipid than sonication, and saponification failed to detect significant amounts of residual bound lipids, the single-step bead beating method was chosen to compare the total lipid yield and composition of all the sub-Antarctic strains and the control strain *C. glutamicum* (Table 2). The major lipid classes found in each bacterial lipid extract were quantified against the calibration curves (Figure 2), with bacterial extracts appropriately concentrated or diluted in chloroform to fit within the range of peak areas for each standard curve. Based on total peak area, *R. qingshengii* 1139 was the highest total lipid-producing strain. However, based on the total lipid weight (sum of mg for all of the major lipid classes derived from 20 mg cell biomass), *Williamsia* sp. 1135 produce the highest amount of lipid per cell biomass (31.4%) and *Rhodococcus* sp. 1168 was the highest TAG producer. The polar lipids close to the origin and the distal non-polar lipid were the two major lipid classes synthesized by all bacterial strains by weight under the growth conditions used.

## 3. Discussion

While there are a number of analytical approaches available for detecting and quantifying the relative proportions of MA in actinobacterial lipid extracts, notably chromatography coupled with MS [28,37,46], there is no single method that provides information on all lipid classes present. Quantitation of the total amount of MA has been reported based on different methodologies, including the separation of FA- and MA-methyl esters by TLC, then determining the level of isotopically labelled lipids using phosphorimaging to calculate molar ratios of the two lipid classes [36] and spectrophotometically by forming MA-fuchsin dye complexes [47]. In the current study, we demonstrated that a single development of silica-coated Iatroscan rods using an H:D:A solvent (80:20:0.1, *v/v/v*), originally described by Parrish [43], could separate non-polar, storage (TAG) and polar lipids in standards and bacterial extracts prepared from six sub-Antarctic *Corynebacteriales* strains and MA-producing controls. The scan time for the major non-polar lipid class detected by TLC-FID did not coincide with that of the commercially supplied free MA standard prepared from bovine *M. tuberculosis*. However, NMR and ESI-MS analyses demonstrated MA signatures and structures in the non-polar lipid eluted from the rods, confirming that the major peak contained MA structures. We also suggest that the MA is bound, at least in part, to carbohydrate molecules originating in arabinogalactan structures in the cell wall. This conclusion was based on observing anomeric proton shifts of around δ 5 in the TOCSY experiment for the TDM standard (Figure 8b), which is indicative of the protons in a system corresponding to the MA bound to trehalose and demonstrated that NMR could detect MA linked to carbohydrate despite the low signals. NMR of the purified non-polar lipid from strain 1139 detected resonance shifts mainly in the region δ 3.5–4.1, which corresponded to clustered signals as expected for D-galactose and L-arabinose. Lee et al. [48] identified 10 types of signals in the range between δ 3.81 and δ 5.14 for mycobacterial arabinogalactan in NMR spectra which depended on the composition of glycosyl linkages. As the peak area of the non-polar lipid was very broad in each of the TLC-FID chromatograms for all bacterial extracts, there may be a range of MA-bound compounds present. Other unidentified lipid compounds may also co-elute with bound MA, the nature of which needs further investigation. Free MAs were detected by UPLC-MS/MS analysis in a non-polar lipid eluted from rods, and it is possible that MAs linked to sugars may have undergone in-source, collision-induced dissociation (IS-CID) within the electrospray interface, yielding free MAs [49]. As the size of the signals for these putative carbohydrate-related protons in NMR was a small proportion of the total seen for the MA signals, it was possible to quantify total MA in samples using the WE standard, which migrated with a similar scan time to MA, as the surrogate standard.

Accurate quantification of lipids by TLC-FID requires minimizing inter- and intra-day variation in analysis arising from sample preparation and storage (avoiding derivatization and oxidation), the application of samples onto rods, the use of different batches of rods and FID detection [41,44]. We controlled the operating procedures of the Iatroscan MK-6s instrumentation by using optimal running conditions (hydrogen and air flow, rapid FID scan time) [44], auto-loading rods with a standard 1 µL sample and pre-scanning rods by FID prior to loading replicates of standards and samples. Bead-beating bacterial cells in the presence of the chloroform:methanol (2:1) solvent to extract lipids from both surface and intracellular locations of the sub-Antarctic strains proved to be more effective than sonication, as saponification of cell debris after bead beating failed to extract significant amounts of structural or storage lipids. Hsu et al. [28] used a similar approach to extract unbound MA from *R. equi* using the same solvent, extracting the remaining bound MA after sonication and saponification of the cell debris. They obtained a greater quantity of covalently bound MA than unbound MA based on the signal to noise ratio of ESI-MS mass spectra. We observed similar trends with *R. qingshengii* with sonification but noted that sonication failed to release more lipids than bead beating. Similar approaches have been used by other researchers dealing with microbes with tough cell wall structures. Byreddy et al. [50] extracted maximum lipid quantities from *Thraustochytrid* strains (heterotrophic fungus-like clades of *Stramenopiles*) using chloroform:methanol (2:1) compared to other solvent systems but sonication worked better than bead beating. In the case of microalgae, Baldev et al. [51] improved lipid recovery using bead beating over sonication, but maximum yield was obtained using grinding followed by bead beating. These observations indicate that relatively harsh cell lysis methods coupled with appropriate solvent extraction are needed to maximize lipid release for analysis from microbes with complex cell walls.

TLC-FID is 2–3 times less sensitive than GC-FID and reduced sensitivity is one of the major drawbacks of this system [48]. Furthermore, TDM and PL standards had similar scan times in the H:D:A (80:20:0.1, *v/v/v*) solvent system, so when TDM occurred in the extracted lipids, as observed for the *M. phlei* control, split or broad peaks were detected, which made quantitation of each lipid class difficult. Increasing the polarity of the eluting solvent by adding more diethyl ether and adjusting the proportion of hexane in the existing solvent system may help to separate these lipid classes [52]. Separation of all the lipid classes in a single step development in TLC-FID is difficult [40] and protocols usually involve multistep development and partial scanning to separate complex lipid classes [38,40]. For example, Striby et al. [41] proposed six hexane-based solvent systems to separate and quantify 17 lipid classes in marine samples. There were relatively fewer lipid classes detected in the *Corynebacteriales* extracts tested and these were clearly separated using a single development method, providing a simple approach to detecting and quantifying these.

Some *Corynebacteriales* species are well documented for TAG production as a major storage lipid. However, this is dependent on bacterial strain and culture conditions [53]. For instance, Alvarez et al. [54] demonstrated that *R. opacus* PD630 accumulated TAG as major inclusions together with some DAG and FFA, which constituted up to 76% and 87% of cellular dry weight when grown on gluconate or olive-oil, respectively, whereas *R. rhodochrous* accumulated both TAG and WE when cultured in media with glucose as the main carbon source [55]. In the experiments presented in the current study, TAG was identified as a major component of the lipid profiles of some of the sub-Antarctic species tested but there was no evidence of WE synthesis, as shown in other rhodococci species which make detectable but proportionately low quantities of WE [56]. However, TAG was a relatively small proportion of the total lipids detected in the sub-Antarctic strains, possibly because the growth conditions were not optimized for TAG synthesis. For these experiments, strains were grown in shake flasks with 1% D-fructose as a preliminary screen for finding the highest lipid-producing strains and for detecting any TAG accumulation. Compared to other strains tested here, *Rhodococcus* sp. 1168 and the two *Williamsia* strains are candidates for potential production of large amounts of TAG. However, the culture conditions for each strain will need to be optimized to maximize lipid production.

Although it has some drawbacks in terms of sensitivity and the need for careful standardization of procedures for quantitation [44], TLC-FID is a simple, comprehensive and rapid lipid-class-screening tool, which can separate lipid classes by adjusting the solvent polarity and this is achieved without saponification, transesterification or any derivatization of extracted lipids [38,43]. Lipid extraction by extensive bead beating cells in chloroform:methanol proved useful, as most of the lipids, including bound and unbound MA, were released from cells in a single step without saponification. Compared to other available methods, we concluded that TLC-FID is a useful platform for the rapid screening and quantification of lipid classes in *Rhodococcus* and *Williamsia* cells when handling multiple samples during optimizing culture conditions for lipid synthesis.

## 4. Materials and Methods

### 4.1. Bacterial Strains and Media

Six *Corynebacteriales* strains originally isolated from Macquarie Island and maintained in the Australian Collection of Antarctic Microorganisms (ACAM) were previously identified by whole-genome sequencing [57,58,59]. These included two novel *Williamsia* sp. strains (1135 and 1138), *R. qingshengii* 1139, *R. erythropolis* 1159 and two novel psychrophilic *Rhodococcus* sp. strains 1163 and 1168. *Corynebacterium glutamicum* and *Mycobacterium phlei* were obtained from the culture collection of the Tasmanian Institute of Agriculture, University of Tasmania, Australia, and used in this study as control strains known to make well-characterized MA.

The six sub-Antarctic strains were grown in minimal salt media (MSM) containing: 7 g/L K_2_HPO_4_; 2 g/L KH_2_PO_4_; 0.25 g/L sea salt (Instant Ocean, Blacksburg, VA, USA); 2 g/L NH_4_H_2_PO_4_ as nitrogen source; 10 g/L D-fructose as carbon source; 0.01 g/L FeSO_4_ and 10 mg/L thiamine hydrochloride (Sigma-Aldrich, Castle Hill, NSW, Australia). The salts plus nitrogen source solution were autoclaved and the carbon source, FeSO_4_, and thiamine hydrochloride supplemented from a 20% (*w*/*v*), 10% (*w*/*v*) and 1% filter sterilized (0.22 µm CA syringe filter, MicroScience, Taren Point, NSW, Australia) solutions, respectively. The pH was adjusted to 7.0 with sterile NaOH after supplementation if required. Cells were cultured at 25 °C in conical flasks agitated at 200 rpm. *C. glutamicum* and *M. phlei* were grown in Brain Heart Infusion broth at 37 °C (Sigma-Aldrich, Castle Hill, NSW, Australia).

### 4.2. Cell Lysis by Bead Beating and Sonication

To compare the lipid extraction efficiency between bead beating (method developed in this study) and sonication described by Hsu et al. [28], 20 mg samples of freeze-dried stationary-phase cells of *R. qingshengii* 1139 grown in MSM broth were transferred to 1.5 mL microcentrifuge tube (Eppendorf, Macquarie Park, NSW, Australia) in three replicates for each method. Chloroform:methanol (2:1, *v/v*) (800 µL) was added into each tube. For bead beating, 1 g of 0.1 mm Zirconia/silica beads, (Daintree Scientific, St Helens, TAS, Australia) was added into the tubes. Both bead beating (30 cycle/min) and sonication were performed for 6 min with 1 min intervals on ice for bead beating, and at room temperature for sonication, using a TissueLyser II (Qiagen, Germantown, MD, USA) and Microson^TM^ ultrasonic cell disruptor (Misonix, Farmingdale, NY, USA), respectively. Tubes were left for 1 h at room temperature and then centrifuged at 15,000×*g* for 5 min; supernatants (the unbound lipids) were collected. Cell debris was extracted five times with 800 µL chloroform:methanol (2:1, *v/v*). The combined supernatant fluids were evaporated under a nitrogen stream at 50 °C and the residue dissolved normally in 500 µL of chloroform, or 250 µL where noted to concentrate lipids for further analysis. To extract any remaining cell-wall-bound lipids, the cell debris was dried under a nitrogen stream and saponified with 4 mL of 10% KOH (w/v) at 80 °C for 1 h in 7 mL Teflon-lined sealed tubes (Sigma-Aldrich, Castle Hill, NSW, Australia), cooled to room temperature, acidified to a pH of approximately 2 with concentrated HCl, and extracted with 2 mL of hexane three times [28]. After evaporating the solvent as described above, the lipids were dissolved in 250 µL of chloroform. The other bacterial strains were subsequently bead beaten and lipids were extracted as described above.

### 4.3. Separation of Lipid Classes Using Plate TLC

TLC was used to detect bound and unbound lipids in bacterial extracts obtained by bead beating. All lipid standards were purchased from Sigma-Aldrich (Castle Hill, NSW, Australia). Lipid reference standard (Supelco mono-, di- and tri-glycerol mix, 1787-1AMP) and individual lipids (MAG, DAG, TAG, FFA, HC, β-hydroxy palmitic acid and MA from bovine *M. tuberculosis*) were dissolved in chloroform to achieve 10 mg/mL for each lipid or used at the supplied concentration (3.9 mg/mL for the PL standard). Lipid solutions were spotted (2 µL) onto preparative aluminium baked silica gel plates (Sigma-Aldrich, Castle Hill, NSW, Australia) and separated using hexane:diethyl-ether:formic acid (H:D:F) (70:30:0.2, *v/v/v*), a solvent similar to one described originally by Striby et al. [41], to resolve TAG, FA and DAG isomers using TLC-FID. Lipid spots were detected by spraying plates with 10% (w/v) molybdophosphoric acid (ethanoic solution), followed by 15 min heating at 120 °C.

### 4.4. Separation of Lipid Classes Using TLC-FID

An H:D:A (80:20:0.1, *v/v/v*) solvent, based on procedures described by Parrish [43] to separate lipid mixtures including WE and TAG, was routinely used to separate and quantify the bacterial lipid classes. The separation efficiency of 70:10:0.1 (*v/v/v*) H:D:A was also assessed. Immediately before loading samples, silica-coated Iatroscan rods (CHROMAROD-S5, SES GmbH Analysesysteme, Bechenheim, Germany) were ‘blank scanned’ in the flame-ionization detector (FID) (IATROSCAN MK-6s, SES GmbH Analysesysteme, Bechenheim, Germany) using 160 mL/min hydrogen and 2 L/min air, to remove all organic impurities and to optimize the activation conditions, as instructed in the instrument’s manual (http://www.ses-analysesysteme.de/IATROSCAN_MK_6_ProcedureUK.htm). Lipid extracts and standards, WE, TDM (Sigma-Aldrich, Castle Hill, NSW, Australia) and the above-mentioned individual standards, were spotted (1 µL) onto Iatroscan rods at lipid concentrations between 1 and 10 mg/mL (1–10 µg loaded) using a semi-automatic injector (SES GmbH Analysesysteme, Bechenheim, Germany), then rods were developed in a covered tank for 30 min with 80 mL of solvent mixture. The tank was prepared at least 30 min before developing the samples and a filter paper was used to assist in tank solvent equilibration. Rods were then dried in a 60 °C oven for 10 min and scanned with the FID at 160 mL/min hydrogen, 2 L/min air and scan speed of 0.5 min/rod [41,44]. i-ChromStar software (http://ses-analysesysteme.de/chromstar_UK.htm) was used to analyze the data. Chromatograms represent FID scans from left to right and rods were developed right to left. All analyses were performed in triplicate and extractions performed at least in duplicated for each strain tested. Representative chromatograms are presented in the reported data.

### 4.5. GC-MS Analysis to Identify Non-polar Lipid Peaks in Bacterial Extracts

A large non-polar lipid peak was observed in the TLC-FID chromatograms of all extracts of test and control bacterial strains, where the scan time coincided with the HC and WE standards. To determine whether these peaks contained HC and/or WE, or other cellular lipids, neutral lipids were extracted from bacterial cells as described by Nichols et al. [39]. Freeze-dried cells (10 mg) were taken into 15 mL glass vials, 2 mL deionized water, then 5 mL methanol and 2.5 mL chloroform added and mixed vigorously, and vapor was vented. This mixture was kept overnight for lipid extraction before adding 2.5 mL of chloroform and 2.5 mL of water, mixing gently and allowing the two phases to separate. The lower organic phase was taken out with a Pasture pipette into a new sealable test tube, then 1 mL of methanol was added. The lipid extract was evaporated to dryness and 3 mL of 5% KOH (*w*/*v*) solution in 80:20 (*v/v*) methanol:water was added. The sealed tubes were heated to 80 °C for 3 h. This solution was allowed to cool, and 1 mL distilled water was added. The neutral lipids (HC and FFA/alcohols released from the saponified wax esters) were extracted twice with 1 mL of hexane:chloroform (4:1, *v/v*) and collected into a GC vial. Any alcohols and FFA present were converted to their TMS derivatives prior to analysis by the following procedure. The extracts were evaporated to dryness under a stream of nitrogen, and reacted with 50 µL of N,O-bis(trimethylsilyl)-trifluoroacetamide solution (Sigma-Aldrich, Castle Hill, NSW, Australia) containing 1% (*v/v*) trimethylchlorosilane and 50 µL of chloroform for 3 h at 50 °C. After cooling, and evaporating to dryness under a stream of nitrogen, samples were made to volume in chloroform for GC-MS analysis.

Neutral lipids were analyzed using a Varian 3800 GC coupled with a Bruker 300 triple quadrupole MS fitted with a 30 m × 0.25 mm Agilent VF-5 ms column (0.25 µm film thickness, Agilent Technologies, Santa Clara, CA, USA). Sample solutions were injected in the splitless mode. The injector and the transfer line temperature were kept at 300 and 290 °C, respectively. Helium was used as the carrier gas at a flow rate of 1.3 mL/min. The oven temperature program was set as: initial temperature 50 °C for 1 min, then increased at rate of 30 °C/min to 150 °C, 2 °C/min to 250 °C and then 5 °C/min to 320 °C, which was held for 5 min. GC-MS operation was controlled by Star software. Full-scan MS spectra in the range of 40–450 (m/z) were obtained. For reference to individual HC TMS-fatty acids and TMS-alcohols, mass spectra were compared to the NIST database (NIST/EPA/NIH Mass Spectral Library 2017).

### 4.6. Identification of Non-Polar Lipids Using UPLC-MS/MS and NMR

To identify the non-polar lipids, a bacterial extract from *Williamsia* sp. 1138 was applied to six Iatroscan rods. After development using the solvent system H:D:A (80:20:0.1, *v/v/v*) the areas corresponding to the unknown non-polar lipids (scan time 0.11 min) were washed from the rods with methanol. Washed rods were heated at 60 °C for 10 min and rescanned using FID to evaluate retained lipids. Methanol washes were pooled then concentrated under nitrogen flow and MA were identified by UPLC-MS/MS in negative electrospray ionization (ESI) mode using a Waters Acquity H-Class UPLC instrument coupled to a Waters Xevo MS/MS (Waters Corporation, Milford, MA, USA). For MA analysis, the electrospray needle was set at 2.8 kV. The ion source temperature was 130 °C, the desolvation gas was N_2_ at 950 L/h, the cone gas flow was 100 L/h and the desolvation temperature was 450 °C. MA molecular species were detected in full scan mode experiments over the mass range (m/z) 400 to 1000 with a cone voltage of 60 V. Data were processed using MassLynx software (version 4.1, Waters Corporation, Milford, MA, USA). A Waters Acquity UPLC BEH C18 column (2.1 mm × 100 mm × 1.7 µm) was used (Waters Corporation, Milford, MA, USA). The solvents used for UPLC consisted of solvent A acetonitrile containing 1 mM acetic acid while solvent B was isopropanol:hexane (8:2, *v/v*). Initial conditions were 100% solvent A for 1 min, before a gradient elution to 100% solvent B over 14 min, which was held for 5 min before returning to initial conditions and equilibrating for 3 min. Total flow was 0.3 mL/min.

To obtain a more concentrated extract of non-polar lipids, 2 µL of bacterial extract (*R. qingshengii* 1139) was applied to each of 10 lanes of a TLC plate and 2 µL of rod-washed material applied as the control to determine where the non-polar lipid ran when eluted with the H:D:A (80:20:0.1, *v/v/v*) solvent. The TLC lane with the rod-washed non-polar lipid was developed by spraying 10% molybdophosphoric acid. Target spots from the bacterial extracts were identified by their migration distance equivalent to the rod-washed material. Silica gel corresponding to these spots was collected from 10 lanes by scraping the silica and then extracting lipids with 3 × 2 mL hexane:chloroform (4:1, *v/v*). The combined extracts were passed through chloroform-prewashed, cotton wool-plugged Pasteur pipettes to remove remaining solids, then evaporated under nitrogen flow and re-dissolved in 500 µL of chloroform. The presence of non-polar lipids in the final extract was reconfirmed by TLC-FID and the extract was then analyzed by NMR, in parallel with commercial free MA and TDM standards.

NMR samples were dried under nitrogen gas and reconstituted in 500 µL of 99.9% CDCl_3_ (Cambridge Isotope Laboratories, Tewksbury, MA, USA) in 5 mm NMR tubes (600 MHz rated borosilicate) (Bruker Pty Ltd., Preston, VIC, Australia). 1D ^1^H spectra were recorded on a Bruker Avance III HD spectrometer at 300 K in a 5 mm TCI cryoprobe; 64 K datapoints were recorded over a spectral width of 20 ppm centered at 6.17 ppm. The number of transients recorded ranged from 16–128 scans based on relative sample concentration. Spectra were referenced indirectly to DSS (4,4-dimethyl-4-silapentane-1-sulfonic acid) at 0.0 ppm and internal CHCl_3_ resonance at 7.27 ppm. Data were zero-filled to 128 K datapoints, a line broadening of 0.3 Hz was applied, and Fourier transformed, phase corrected, and baseline corrected, all using Topspin 3.5.b91-pl7 (Bruker Pty Ltd., Preston, Victoria, Australia). 2D multiplicity edited ^13^C-^1^H HSQC (Heteronuclear Single Quantum Correlation) and ^13^C-^1^H HMBC (Heteronuclear Multiple Bond Correlation) and ^1^H-^1^H COSY (Correlation Spectroscopy) and TOCSY (Total Correlation Spectroscopy) experiments were also recorded and variously processed to provide context to analysis of 1D data for the TDM control. All experiments were run using standard pulse programs in the Bruker Avance III HD spectrometer library. 1D data were compared with published chemical shifts documented for MA structures [45] and ^1^H-NMR spectra for sugars (Human Metabolome Database, http://www.hmdb.ca, accessed February and May 2019).

### 4.7. Preparation of the TLC-FID Calibration Curve to Quantify Lipid Classes

Mixtures of selected standards (MA, FFA [nonadecanoic acid, C19], PL, WE, TAG) in chloroform were prepared, where each compound was at 1, 2, 3 or 4 mg/mL and 1 µL of each mixture was spotted onto triplicate Iatroscan rods. Conditions for separation, scan-time and peak area measurement are described above. Standard curves were prepared from non-linear regression equations for power trend lines for each lipid standard using Excel. To quantify the non-polar lipid observed in bacterial extracts, WE was used as a surrogate standard and concentrations were calculated against the WE calibration curve. The polar lipid peak close to the origin was calculated against the PL standard calibration curve.

## Figures and Tables

**Table 1:** Comparison of total lipid peak area and major lipid classes of *R. qingshengii* 1139 obtained by bead beating and sonication and detected by TLC-FID.

Lipid Extraction Methods	Total Peak Area (µV/s)	Detectable Lipid Classes (mg/500 µL) by TLC-FID						Lipid % of Cell Dry Weight
		NPL	TAG	FFA	MA	PL/TDM	Total	
Bead beating (unbound)	24410	2.20	0.37	0.69	-	2.45	5.71	28.6
Bead beating (bound) ^a^	284	-	-	-	-	-	-	1.2
Sonication (unbound)	11789	1.24	0.26	0.59	-	1.50	3.59	18.0
Sonication (bound) ^a^	5098	0.49	-	0.97	-	0.37	1.83	9.2

^a^ Lipid extracted from saponified cell debris after extraction of unbound lipids.

**Table 2:** Total lipid peak area and major lipid classes detected in extracts of five sub-Antarctic strains and control strain detected by TLC-FID following bead beating.

Strains	Total Peak Area (µV/s)	Detectable Lipid Classes (mg/500 µL) by TLC-FID						Lipid % of Cell Dry Weight
		NPL	TAG	FFA	MA	PL/TDM	Total	
*Williamsia* sp. 1135	23042	1.91	0.66	0.67	0.84	2.2	6.28	31.4
*Williamsia* sp. 1138	21250	2.01	0.60	0.66	-	1.60	4.87	24.4
*Rhodococcus* sp. 1159	22228	1.89	0.44	-	-	2.61	4.94	24.7
*Rhodococcus* sp. 1163	16343	1.34	0.44	0.65	-	2.09	4.52	22.6
*Rhodococcus* sp. 1168	16151	1.57	0.78	0.64	-	1.60	4.59	23.0
*C. glutamicum*	14697	1.71	-	-	-	1.71	3.42	17.1

## Supplementary Materials

Supplementary materials can be found at https://www.mdpi.com/1422-0067/21/5/1670/s1.
