# Supplementary material for: Application of Thin-Layer Chromatography-Flame Ionization Detection (TLC-FID) to Total Lipid Quantitation in Mycolic-Acid Synthesizing Rhodococcus and Williamsia Species
Source: Int J Mol Sci. 2020 Feb 29;21(5):1670. doi: 10.3390/ijms21051670 (PMC7084869; doi:10.3390/ijms21051670)
Supplement: Supplementary file 1 [file ijms-21-01670-s001.pdf]

Supplementary Material

A method for total lipid analysis by thin-layer chromatography-flame ionization detection (TLC-FID) in *Rhodococcus* and *Williamsia* species

Akhikun Nahar[1], Anthony L. Baker[1], David S. Nichols[2], John P. Bowman[1], Margaret L. Britz[1,*]

[1] Tasmanian Institute of Agriculture, University of Tasmania, Hobart, TAS 7005, Australia; akhikun.nahar@utas.edu.au; a.l.baker@utas.edu.au; john.bowman@utas.edu.au; margaret.britz@utas.edu.au

[2] Central Science Laboratory, Division of Research, University of Tasmania, Hobart, TAS 7005, Australia; david.nichols@edu.au

* Correspondence: Margaret.Britz@utas.edu.au

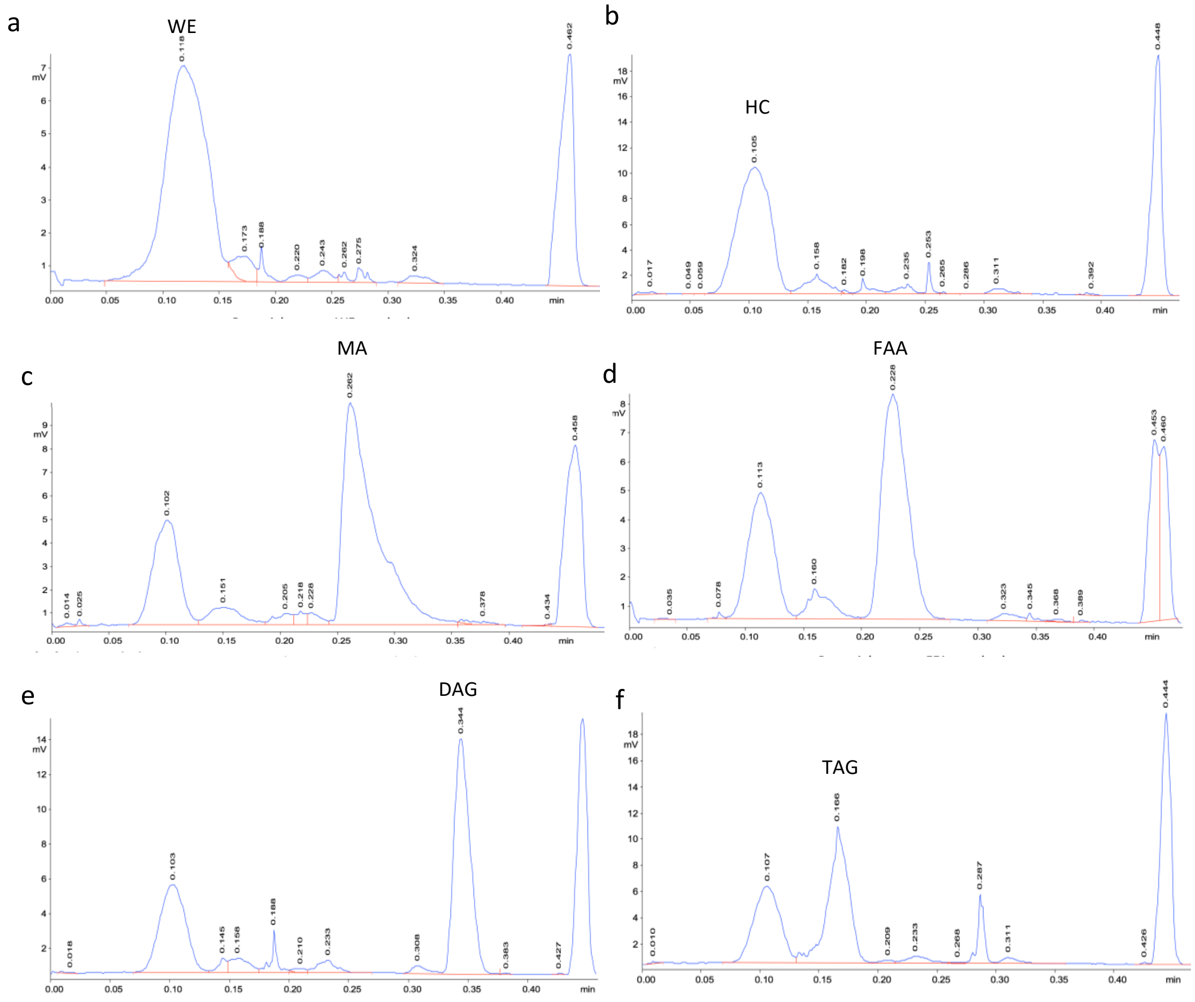

Figure S1. TLC-FID chromatograms of bacterial extract (*Williamsia* sp. 1138) mixed with different standards to demonstrate co-elution of unknown and standard compounds. a) WE, b) HC, c) MA, d) FFA, e) 1,2-DAG and f) TAG. Panels a) and b) show that the retention time of the non-polar lipid peak coincided with the WE and HC standards, respectively. Abbreviations are shown in Figure 2. Panel d) shows a split peak in the region of the PL peak, likely due to monoacylglycerols in the free fatty acid standard A peak near the retention time pf PL Panel c) shows the tailing peak for the mycobacterial free MA standard and a minor merged peak on the side of this region which we suggest corresponds to free MA in strain 1138, noting that

the MA chain length is considerably shorter in the *Williamsia* and *Rhodococcus* strains compared to the standard.

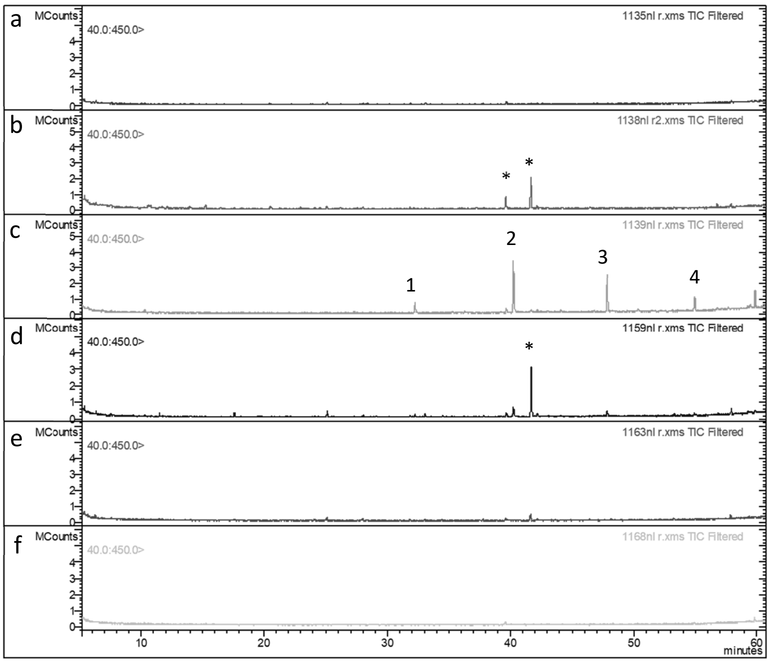

Figure S2. Full scan GC chromatograms of neutral lipid extracts of a) *Williamsia* ap. 1135, b) *Williamsia* sp. 1138, c) *Rhodococcus qingshengii* strain 1139, d) *Rhodococcus erythropolis* strain 1159 (1. Octadecanal, 2. Docosanal, 3. Tetracosanal and 4. Hexacosanal), e) *Rhodococcus* sp. 1163, f) *Rhodococcus* sp. 1168. * = artefact. The absence of non-saponifiable (HC) or saponifiable (alcohol) neutral lipids indicated the absence of WE complexes in the bacterial extracts for these strains although other species of *Rhodococcus* are reported to synthesise WE compounds.
